# The h-Current in the Substantia Nigra pars Compacta Neurons: A Re-examination

**DOI:** 10.1371/journal.pone.0052329

**Published:** 2012-12-21

**Authors:** Cristina Gambardella, Angela Pignatelli, Ottorino Belluzzi

**Affiliations:** Dipartimento di Scienze della Vita e Biotecnologie, University of Ferrara and Istituto Nazionale di Neuroscienze, Ferrara, Italy; Vanderbilt University Medical Center, United States of America

## Abstract

The properties of the hyperpolarization-activated cation current (I_h_) were investigated in rat *substantia nigra - pars compacta* (SNc) principal neurons using patch-clamp recordings in thin slices. A reliable identification of single dopaminergic neurons was made possible by the use of a transgenic line of mice expressing eGFP under the tyrosine hydroxylase promoter. The effects of temperature and different protocols on the I_h_ kinetics showed that, at 37°C and minimizing the disturbance of the intracellular milieu with perforated patch, this current actually activates at potentials more positive than what is generally indicated, with a half-activation potential of −77.05 mV and with a significant level of opening already at rest, thereby substantially contributing to the control of membrane potential, and ultimately playing a relevant function in the regulation of the cell excitability. The implications of the known influence of intracellular cAMP levels on I_h_ amplitude and kinetics were examined. The direct application of neurotransmitters (DA, 5-HT and noradrenaline) physiologically released onto SNc neurons and known to act on metabotropic receptors coupled to the cAMP pathway modify the I_h_ amplitude. Here, we show that direct activation of dopaminergic and of 5-HT receptors results in I_h_ inhibition of SNc DA cells, whereas noradrenaline has the opposite effect. Together, these data suggest that the modulation of I_h_ by endogenously released neurotransmitters acting on metabotropic receptors –mainly but not exclusively linked to the cAMP pathway- could contribute significantly to the control of SNc neuron excitability.

## Introduction

The presence of the h-current is an hallmark of midbrain dopaminergic (DA) neurons, including those of the substantia nigra pars compacta (SNc), up to the point that its occurrence is considered by many authors the main discriminating criterion to decide if a given neuron in this area is dopaminergic or not [1]. Many studies have confirmed the close relationship between the DAergic phenotype and I_h_ expression [2]–[6]. Apart from the abundant electrophysiological evidence ([7]–[9], to cite a few), the presence of h-channels in SNc neurons is also supported by qualitative RT-PCR experiments on single cells, which revealed that SNc neurons co-express three of the four types of HCN subunits: HCN2, HCN3, and HCN4 [10].

As expected, the presence of a current typically associated with the pacemaking process (see [11] for a review) suggests that it could play its archetypal role also in SNc neurons, cells characterized by autorhythmicity. However, several studies reported that I_h_ has neither a significant role in spontaneous pacemaker activity nor does it contribute substantially to the setting of the resting potential [9], [12]–[15].

Overall, the present knowledge of the h-current in SNc neurons is not entirely satisfactory, and this is all the more surprising for a population of neurons which is object of so many studies. The inconsistencies in the description of I_h_ are probably due to the strong dependence of the kinetics of this current on experimental conditions (e.g., temperature, patch configuration, ionic composition of solutions, modulation by cytoplasmic cyclic nucleotides, protocols used, etc.). This circumstance may explain why, even for a single cell type, different kinetics were found by different laboratories, and consequently different roles were proposed. In addition, there might be a problem in the cell identification: as a rule, cells in the midbrain are identified as dopaminergic on the basis of a series of electrophysiological characteristics, confirming *a posteriori* the identification in few randomly chosen cells with immunohistochemistry to ascertain the presence of TH. However, some of the more commonly used identification criteria are not really discriminative. For example, the presence of I_h_ -considered a benchmark- can be misleading, as if the absence of this current in a midbrain neuron is a trustworthy predictor that the cell is not DAergic, its presence does not reliably predict TH co-labeling [16], [17]. A novelty of this study is in the use of a transgenic line of animals that expresses a reporter protein (eGFP) under the TH promoter, allowing the exact identification of each studied neuron as DAergic.

In this work we first report a kinetic characterization of the h-current in SNc neurons as close as possible to the physiological conditions (temperature, perforated patch), showing that this current at rest is larger than the one usually obtained. Then we describe how the resting membrane potential of the dopaminergic ‘principal’ cells are affected by this current. Finally, we show that neurotransmitters physiologically released onto SNc neurons can modulate the h-current, thereby affecting the overall excitability profile of these cells.

## Results

The results of this study are based on observations made in 357 voltage-clamped SNc neurons, all showing inward rectification at voltages negative to the resting membrane potential.

In order to isolate the h-current, except where otherwise stated, all the other main ionic currents were blocked; in particular, the sodium current with 0.6 µM tetrodotoxin (TTX), the delayed rectifier potassium current with 20 mM tetraethylammonium (TEA), the A-type potassium current with 3 mM 4-aminopyridine (4AP), the calcium and calcium-dependent currents with 100 µM cadmium and the K_IR_ current with 0.5 mM barium.

### Biophysical Properties

In this first section, all the recordings were carried out in slices at 37°C in whole-cell configuration. The input resistance was 413.64±30.17 MΩ (n = 188; 5–95 percentile range 136–651 MΩ), and the access resistance was 12.9±0.3 MΩ (n = 199; 5–95 percentile range 7.9–18.0 MΩ). In normal recording medium containing 2.5 mM-K^+^ the resting membrane potential of SNc neurons was –60.54±0.92 mV (n = 91; 5–95 percentile range −50.2–71.0 mV), calculated as the potential corresponding to the zero injected current in voltage-clamp conditions. About 95% of the neurons fired spontaneously with a frequency of 4.9±0.67 Hz (n = 57, frequency measured in cell-attached mode).

**Figure 1:**
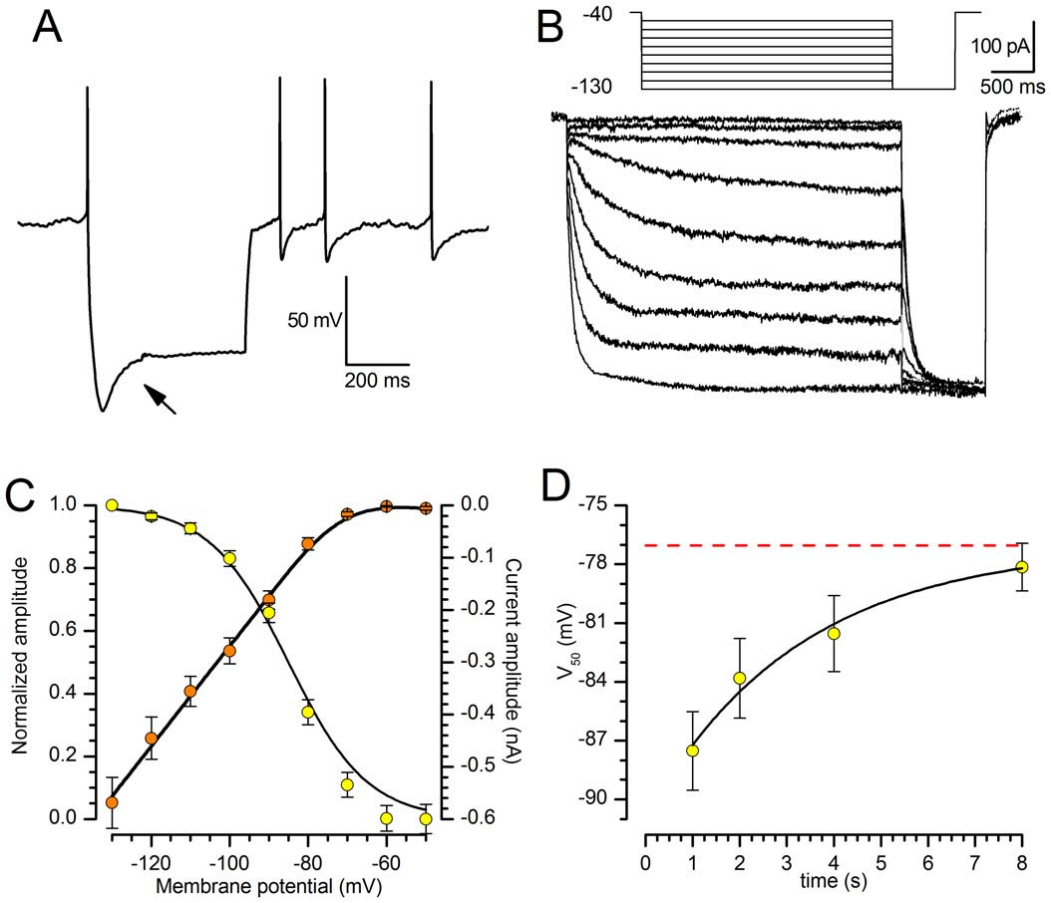
Basic properties of the h-current at 37°C. A: Response of a SNc DA neuron under current-clamp condition to the injection of a 300 pA hyperpolarizing current pulse. Note the appearance of the archetypal sag (arrow) due to the activation of I_h_; resting potential −55 mV, bath solution for this recording was standard ACSF. B: Family of responses of a SN DA neuron under voltage-clamp conditions to the application of the double-pulse protocol shown in the inset above; explanation in the text. C: Current-voltage relationship of the h-current (□, right y-axis) and voltage-dependence of the activation curve (○, left y-axis) obtained from tail analysis of double-pulse experiments as shown in panel B; mean value ± S.E. (n = 20). D: Dependence of the midpoint (V_50_) on the duration of the hyperpolarizing pulse; the dashed line highlights the asymptotical behavior of the midpoint for a conditioning pulse of infinite duration - see text for explanation.

All of the examined SNc neurons showed a time-dependent sag in response to the injection of outward currents (Figure 1A, arrow), becoming evident at potentials more negative than −60/−70 mV; the termination of the current pulses was usually followed by a depolarizing overshoot; the depolarizing sag became progressively bigger as the membrane was stepped to more negative potential.

#### Activation

Under voltage clamp, hyperpolarizing commands from a holding potential of −40 mV evoked slow inward relaxations over the same membrane potential range as the ones producing sags and depolarizing overshoots in hyperpolarizing electrotonic potentials (Figure 1B). The h- current activated slowly and increased the magnitude and rate of activation as the cells were progressively hyperpolarized, with no sign of inactivation. Two current components were measured during the hyperpolarizing voltage steps: (i) an instantaneous current (I_inst_), obtained at the beginning of the step; (ii) a steady-state current (I_ss_), obtained at the end of the step. The instantaneous current was almost linear along the explored voltage, while the steady-state current increased its magnitude as the membrane potential was made more negative; the h-current amplitude, measured as I_ss_-I_inst_ (see methods) is plotted against voltage in Figure 1C (orange symbols).

The steady-state activation curve (Figure 1C, yellow symbols) was obtained by interpolating the relative amplitudes of the tail currents with the Boltzmann function (Eqn. 2, explanation in Methods), finding - for 2 s hyperpolarizing step - values for half-activation (V_50_) of −84.17±1.31 mV (n = 13) and for *k* of 7.74±0.40 mV (n = 13).

The point of half activation of the h-current critically depends on the hyperpolarizing pulse length [18]: conditioning pulses of short duration do not allow the gating process to reach the steady-state condition, therefore the probability of opening can be seriously underestimated, leading to evaluations of V_50_ more negative of their actual value. These measurement errors are more pronounced for slow HCN channels than for the fast ones, and are highly dependent on temperature [19], [20]. Therefore, we have analyzed the dependence of the midpoint from the duration of the conditioning command. In nine cells studied with the double pulse protocol described above, the first command had durations of 1, 2, 4 and 8 s; we also tried the next point in the log scale, 16 s, but the membrane did not withstand the prolonged hyperpolarizations at the more negative potentials. Increasing the duration of the conditioning pulse induces a significant shift of the steady-state activation curves in the depolarizing direction: the values of V_50_ is changed from −87.52±2 mV for 1 s stimuli to −78.15±1.22 mV for 8 s (Figure 1D); we did not observe any change in the corresponding slopes. The V_50_ values as a function of the duration of the first step can be described by the exponential function
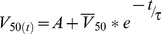
(1)where V_50(*t*)_ is the value assumed by the midpoint of the steady-state activation curve for conditioning potentials lasting *t* seconds; 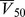 is the range of variation of *V*_50_ as a function of the conditioning period *t*, and has a value of 13.90±1.58 mV; τ is the time constant of the process, 3.21±1.42 s. Finally, *A* is the asymptote (i.e. the value to which *V*_50_ tends for *t* → ∞; dashed line in Figure 1D). The significant value obtained from this analysis is equal to −77.05 mV ±1.54; this means that, once the channel reaches its steady-state conditions, the point of half-activation is about 10 mV more positive than what is generally believed.

#### De-activation

**Figure 2:**
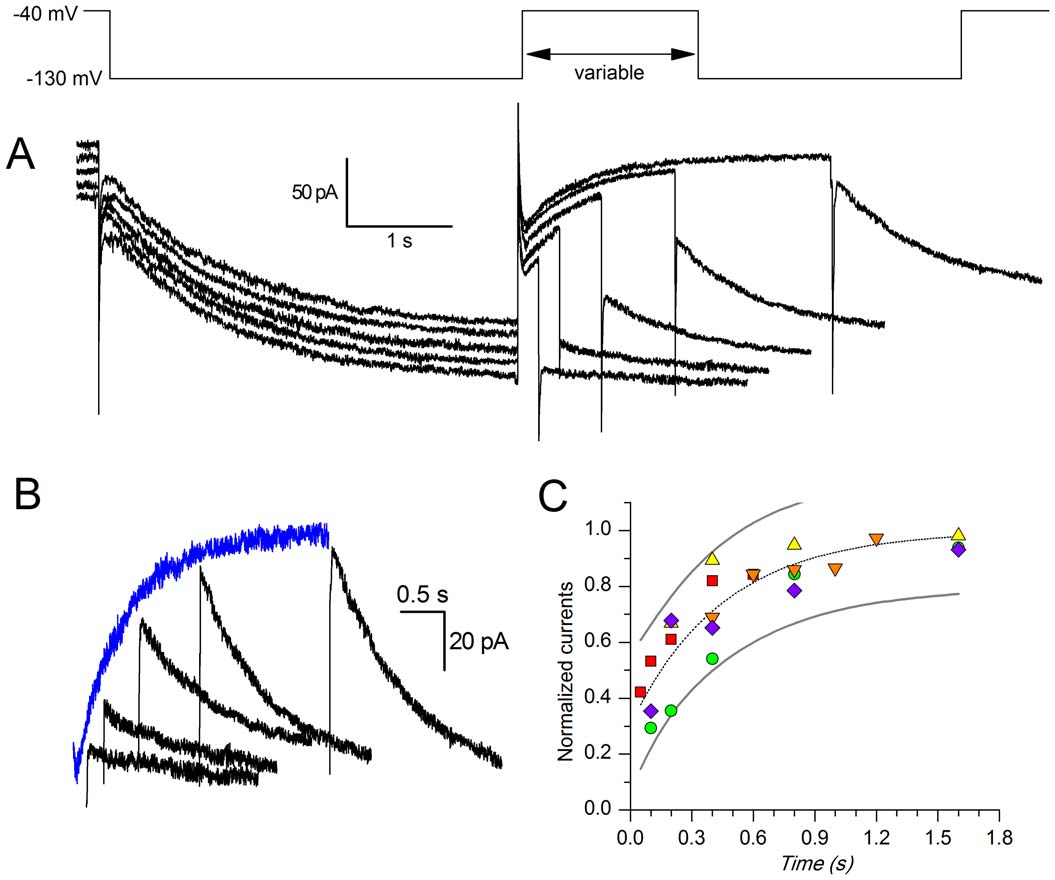
De-activation kinetics. A: Envelope test during deactivation at −40 mV. After current activation at −130 mV, pulses to −40 mV of variable duration were followed by re-activating steps to −130 mV (see protocol in the top panel). B: The tail at −40 mV was also re-plotted after appropriate scaling (grey trace) to better compare its time course with that of the re-activation records envelope shown in panel A. C: Analysis of the deactivation time constant for a group of five cells; the average time dependence (dashed line) was fitted by the equation I_(t) = _1–0.69*exp(-t/0.45) (dashed line; continuous line delimitate the 95% confidence interval).



The de-activation time constant was measured using the envelope test [21] shown in Figure 2: from a holding potential of −40 mV, two hyperpolarizing pulses to −130 mV lasting 4 s were imposed, separated by a repolarization to −40 mV of variable length (Figure 2A). In Fig. 2B, the I_h_ de-activation at −40 mV and the envelope of re-activation records at −130 mV shown in panel A are displayed together, in order to evidence the likeness of their exponential time course. The values of the amplitudes of the tail currents recorded upon re-activation at −130 mV were normalized, plotted as a function of depolarizing step duration (Figure 2C), and the de-activation time constant was calculated by interpolating the experimental points with the exponential function.

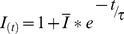
(2)where 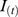
is the normalized current amplitude at time *t*, 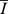 the range of change of the normalized current, τ the time constant of de-activation at the potentials indicated. In a group of 5 cells, τ was 0.45±0.07 s (Fig. 2C).

#### Reversal potential

**Figure 3:**
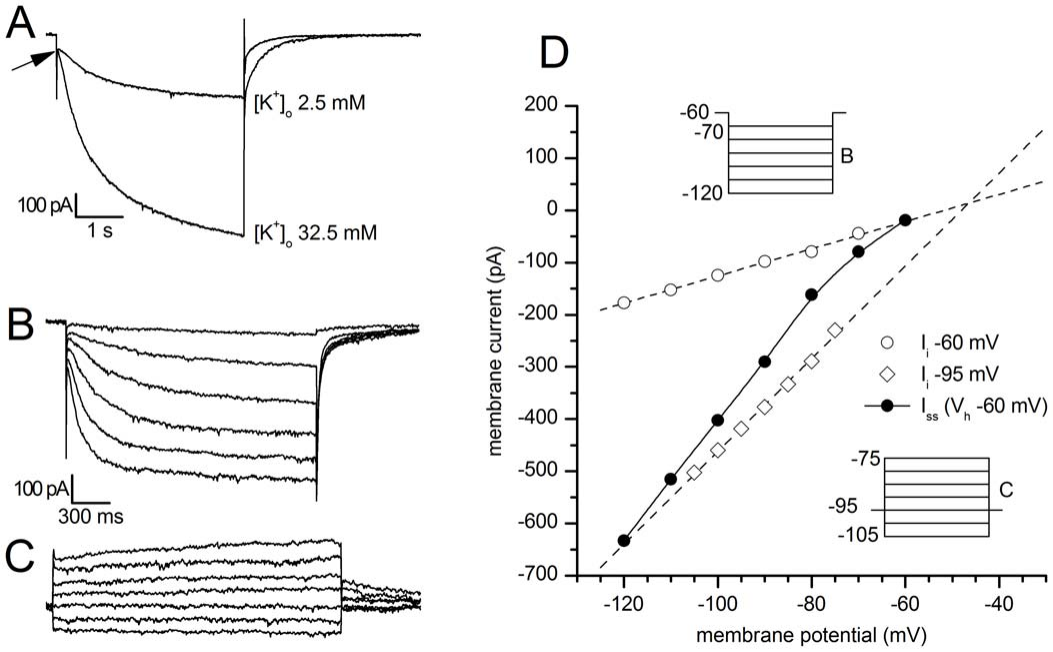
Analysis of Ih reversal potential. A: Response to hyperpolarizing steps from −40 to −130 mV using the indicated concentration of K^+^ ions in the external saline; T = 27°C. B,C: Recordings of the slow current relaxations recorded at holding potentials of −60 and −95 mV, respectively, in response to the indicated protocols represented in the insets of Figure D; T = 37°C. D: Instantaneous (chord) and ‘steady-state’ current-voltage relationships of a SNc DA neuron voltage-clamped at holding potentials of −60 mV (chord conductance 2.60 nS) and −90 mV (chord conductance 8.89 nS). Note that the chord conductance plots are approximately linear at both the holding potentials, despite the presence of strong inward rectification in the ‘steady-state’ current-voltage relationship measured at the end of 2 s hyperpolarizing voltage jumps from a holding potential of −60 mV. Inward rectification in the ‘steady-state’ current-voltage relationship is entirely accounted by these slow relaxations.

The h-current is carried by cation channels permeable to Na^+^ and K^+^ ions [22]–[24]; in fact, increasing the extracellular concentration of potassium from 2.5 to 32.5 mM produced a reversible increase in the amplitude of I_h_. The mean amplitude in I_h_ during exposure to 32.5 mM K^+^ was 476% ±40 of control (n = 5, Figure 3A).

As shown in Figure 3A (arrow), we failed to observe any increase in the instantaneous current in high K^+^ [9]. The explanation for this discrepancy is complicated by the fact that the nature of I_inst_ is not well defined yet [25], contrary to the slower component, which is certainly sustained by cations passing through the well-characterized pore of HCN channels. In addition, I_inst_ usually has a small amplitude, and is not observed in any measurement of I_h_. Speculations on the nature of I_inst_ range from models where this current represents a leak conductance or an experimental artifact, to models in which I_inst_ is caused by a second pore within the same HCN or a second channel population associated with HCN channels [26]–[28]. Midbrain DAergic neurons also have another hyperpolarization-activated current, K_IR_ type, that could contribute to the I_inst_ amplitude, and which is enhanced by an increase of the external K^+^ concentration. It cannot be excluded, therefore, that the difference in the results might be consequence of different degrees of blockage of the K_IR_ current, in addition to possible differences in the animal species used (mouse and rat).

The classical procedure to calculate the reversal potential of a voltage sensitive conductance is from the tail currents reversal, but in SNc neurons this method was rather problematic due to the activation of several outward rectifiers in the membrane potential range over which reversal was expected.

For a more precise calculation of the I_h_ reversal potential (E_h_) we used the method of the instantaneous (chord) and ‘steady-state’ current-voltage relationships [29]. At the membrane potential of −95 mV g_h_ is strongly activated (Figure 3C) and does not show time-dependent inactivation; the reversal potential of I_h_ is then obtained from the intersection of the instantaneous (chord) current-voltage relationships recorded at holding potentials of −60 mV and −95 mV (Figure 3D), i.e. in the absence and presence of I_h_.

In 8 neurons the mean value obtained for the reversal potential at 37°C was −44.03±3.10 mV (range −29.4 to −56.9 mV).

#### Effect of temperature

**Figure 4:**
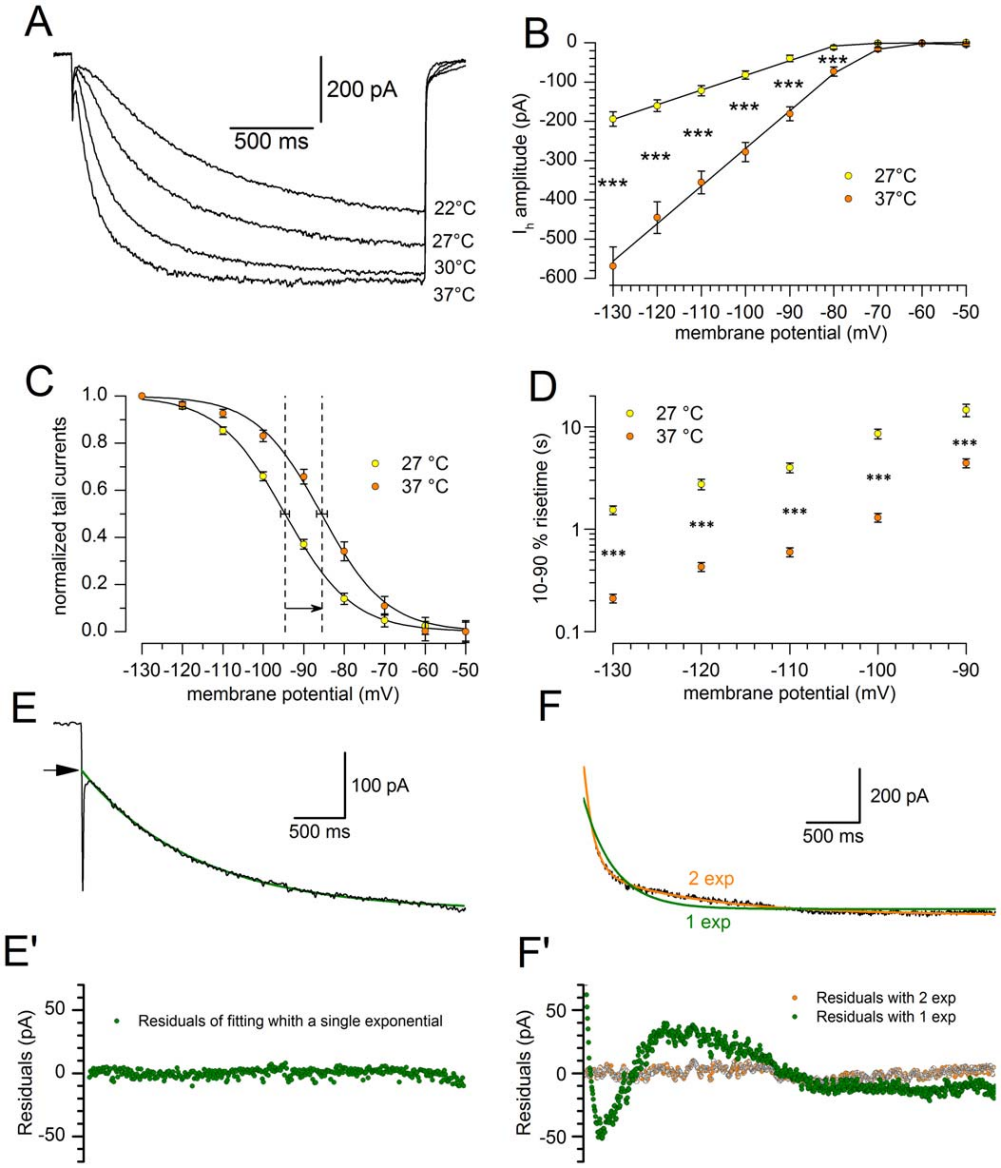
Effect of temperature on h-current amplitude and kinetics. A: Family of current tracings recorded in a single cell in response to hyperpolarizing pulses from −40 to −130 mV, repeated at the temperatures indicated. B: Comparison of the I/V curves recorded at 27 (yellow dots) and 37°C (orange dots); n = 20; the difference, tested with two-way ANOVA and post-hoc Bonferroni test, is significant at 0.001 level for the potentials more negative than −70 mV. C: Shift of the steady-state activation curves for a change from 27°C (yellow) to 37°C (orange), average values from 13 cells ± S.E for the V_50_. D: Effect of the temperature on the h-current 10–90% risetime at 27°C (yellow) and 37°C (orange); see explanation in the text. E, E′: Sample fit with a single exponential of an h-current tracing obtained at 27°C in response to a voltage step to −130 mV; below the analysis of the residuals (experimental data minus the corresponding values of the fitting curve). F, F′: Sample fit with both single (green) and double (orange) exponential of a h-current tracing obtained at 37°C in response to a voltage step to −130 mV; below the analysis of the residuals in the same grey scale code. All the recordings shown in this figure were obtained in perforated patch conditions.

It has been shown in various types of preparation that the kinetics of I_h_ is particularly sensitive to thermic conditions [20], [30], [31]. The temperature at which electrophysiological recordings are made, affecting both the amplitude and the kinetics of I_h_ (Figure 4A), is one of the limiting factors in comparing the results; therefore, in this study most of the reported recordings were realized in precisely controlled temperature conditions.

Figure 4B shows the effect of a 10°C temperature increment on the I_h_ amplitude at different potentials. I/V graphs show the mean current amplitudes recorded at 27°C (•) and 37°C (○) as a function of membrane potential. At −130 mV, a 10°C increase causes a rise in amplitude from −194.2±18.5 pA at 27°C (n = 19) to −569.1±48.9 pA at 37°C (n = 23). The average value of Q_10_ for I_h_ amplitude between −80 and −130 mV is 3.76±0.53. The resulting maximal conductance g_h_ at 27 and 37°C is 2.26 and 6.62 nS, respectively.

We next explored whether the increase of I_h_ at −130 mV could be explained by a shift in the voltage dependency. As seen from the graph (Figure 4C), the transition from 27°C (•) to 37°C (○) causes a shift of the steady-state activation curve by about +10 mV: the V_50_, calculated fitting the Boltzmann equation to the experimental points (4 s conditioning pulses), is −94.91±1.72 mV at 27°C (n = 13) and −84.23±1.28 mV at 37°C (n = 18), (P<0.0001, two-tailed Student *t* test for unpaired data). No significant changes were observed in the steepness of the relationship: the slope is 8.02±0.37 mV at 27°C and 7.74±0.37 mV at 37°C.

The temperature does not affect only the total conductance of the h-current (Figure 4A) but -and to a much higher degree- also its activation kinetics, which is modified under two aspects. First, the tracings at 27°C can be satisfactorily fitted by a single exponential (Figure 4E, E′), but two exponentials are always needed for an adequate fit at 37°C (Figure 4F, F′). Second, the current development rate is strongly affected by the temperature. Since at 27°C there is only an exponential, and at 37°C two, a comparison of the time courses was possible only comparing the 10–90% rise time. Since not always the steady state was reached, due to the instability of the membrane at the more negative potentials, we used the following equations, obtained by solving equation 3 (see methods) for y = 10 and y = 100 after normalization of the amplitude to 100:

- for a single exponential *t_90_* = τ ln(^100^/_10_) and *t_10_* = τ ln(^100^/_90_), where *t_10_* and *t_90_* are the times at which the current is developed for the corresponding percentage, and τ is the time constant;
- for a double exponential the solution was less straightforward: first the amplitudes of the two exponentials (*A_1_* and *A_2_*) were normalized so that their sum was 100; then, Eqn. 1 was solved numerically for *t* [32], [33] setting *f(t)* = 90 and = 10 (the Matlab code used can be found in the Supplementary material as Code S1), obtaining *t_10_* and *t_90_*, respectively.

The comparison of the *t_10_*–*t_90_* times at 27 and 37°C is represented graphically in Fig. 4D, and the corresponding Q_10_, in the range −90 −130 mV, is 6.4, as calculated with Eqn. 3 setting *rate* as (*t_10_*–*t_90_)_._*

The de-activation time constant is also affected by temperature: the double-pulse protocol described above was applied in five cells at 27°C. The normalized amplitudes of the currents to the second pulse as a function of the delay between the two pulses can be described by the exponential function indicated in equation 2, with a time constant of 1.46±0.1 s, which would give a Q_10_ of 3.2 when compared with the value at 37°C indicated in a section above.

### Basic Pharmacology

**Figure 5:**
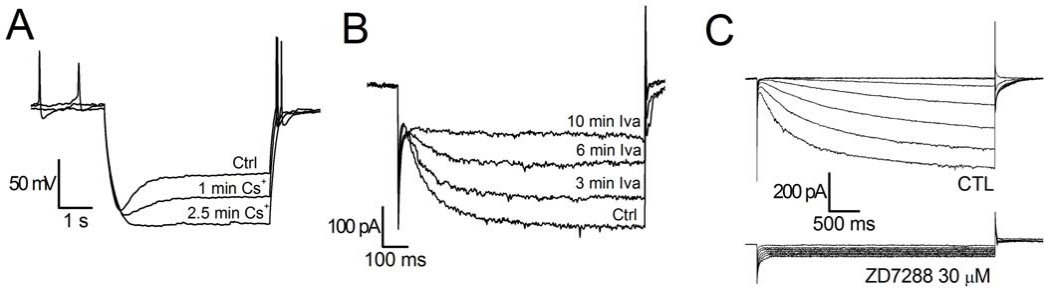
Basic pharmacology of h-current. A: Blockage by Cs+ (1 mM): current-clamp responses to a repeated hyperpolarizing current step of 300 pA from a holding potential of −60 mV. Note the progressive suppression of the sag after the indicated times of Cs+ application; T 37°C. B: Blockage by ivabradine (10 µM): voltage clamp responses to a repeated hyperpolarizing step to −130 mV from a holding potential of −40 mV at the indicated times of drug application; T 37°C. C: Blockage by ZD7288 (30 µM); holding potential −40 mV, test potentials ranging from −70 to −130 mV; T 27°C, Ba2+0.5 mM present throughout.

The h-current is sensitive to low concentrations of Cs^+^ (1–2 mM) [34] and to a certain number of organic compounds blocking selectively the h-channels, like ZD7288 [35] and S-16257 (ivabradine) [36], [37]. Cs^+^1 mM effectively blocks the h-current (Figure 5A). However, as already observed in calf Purkinje cells [38], the action of Cs^+^ is clearly voltage-dependent: in the negative region of the I-V curve Cs^+^ induces a channel blockade, whereas at more positive potentials it is ineffective, and sometimes it can even produce the opposite effect, i.e. a current increase. More selective, and completely voltage-independent blockages, can be obtained with ivabradine 10 µM (Figure 5B) and ZD7288 (30 µM, Figure 5C).

### Role of I_h_ in Autorhythmicity

One of the hallmarks of SN neurons is the autorhytmicity: these neurons fire spontaneously action potentials characterized by an unusually long duration (>2.5 ms), a rather depolarized threshold (> −40 mV) and a marked afterhyperpolarization [1], [39]. The role of the h-current in spontaneous activity has been thoroughly analyzed by several authors, and the conclusion has been that it is neither a significant factor underlying the spontaneous pacemaker activity nor does it contribute substantially to the setting of the normal resting potential level of the membrane [9], [12]–[15].

**Figure 6:**
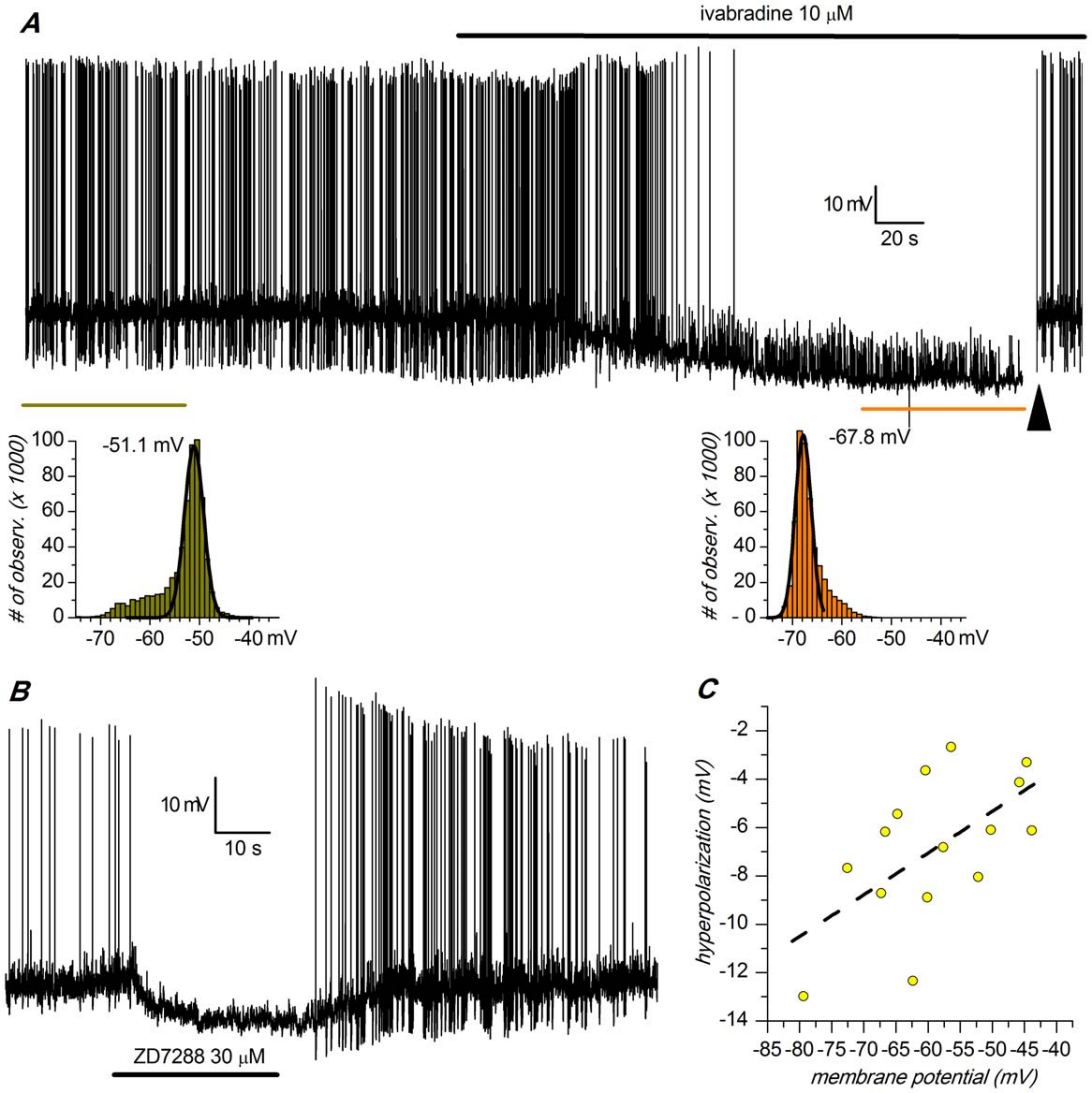
Role of Ih in autorhythmicity. A: Current-clamp recording (perforated patch, 37°C) showing the hyperpolarizing effect of a selective, non voltage-dependent blocker of the h-current (ivabradine 10 µM, applied focally at the moment indicated by the arrow) on spontaneous activity. The two insets below illustrate the method used for the determination of the “resting” membrane potential in spontaneously active cells: the value was actually measured as the prevailing potential, measured by fitting a Gaussian to the membrane potential values in the time intervals marked by the bars of the same color. At the time marked by the large arrow to the right, the membrane was manually depolarized to the value antecedent the ivabradine application, restoring the spontaneous activity and thereby showing that the h-current is not essential for the pacemaking mechanism. B: Same experiment using ZD7288 30 µM. C: Relationship of the hyperpolarizing effects obtained with ZD7288 with the resting membrane potential, showing a significant correlation (p<0.02; ANOVA).

Our data only partially confirm this viewpoint: recording at 37°C and in perforated patches, the block of the h-current by focal application of ivabradine 10 µM does stop the spontaneous activity; the effect is rapid and reversible, and is paralleled by an important hyperpolarization (11.83±2.07 mV, n = 7; Figure 6A). The blockage of spontaneous activity following a membrane hyperpolarization of 10 mV is not surprising, as the cell firing is based on a delicate interplay of conductances [8], [14], [40]–[42] that can be easily disrupted by the injection of outward currents ([43], and personal observation). We then tested whether this blockade represented the evidence for an essential role played by the h-current in the pacemaking mechanism, or if it was only the consequence of the hyperpolarization following the suppression of the h-current. In the presence of ivabradine, the injection of a depolarizing current sufficient to restore the membrane potential to the value antecedent the I_h_ block (arrowhead in Figure 6), resumes immediately the spontaneous activity (Figure 6A, right). This proves the absence of any direct role of the h-current in autorhythmicity, but also demonstrates that this current has a relevant role in determining the membrane potential.

Since the influence of the h-current on membrane potential is somewhat controversial, and since ivabradine is a relatively new drug, for which side effects have been described on currents other than I_h_ [44], [45], we repeated the same experiment using the more classical blocker ZD7288 (30 µM). The results were substantially similar to those obtained with ivabradine (Fig. 6B), although the hyperpolarizing effect was less pronounced (6.87±0.78 mV, n = 15) and in 7 out of 15 cases we were unable to obtain a substantial recovery after 20′ washout. Interestingly, the hyperpolarizing effect of the h-current blockage was correlated with the resting membrane potential (Fig. 6C; p value <0.02, ANOVA), as expected for a conductance whose effect is increasingly influential at more negative potentials.

### Modulation of h-current

#### I_h_ modulation by intracellular cAMP

The h current is dually regulated by hyperpolarization and by cyclic AMP, directly binding to a sequence (cyclic nucleotide binding domain, CNBD) located in the C-terminal segment [46], [47]. We have therefore analyzed the modulatory effect of cAMP on the h-current using a recording configuration (perforated patch with amphotericin B) minimizing the perturbations of the intracellular medium.

**Figure 7:**
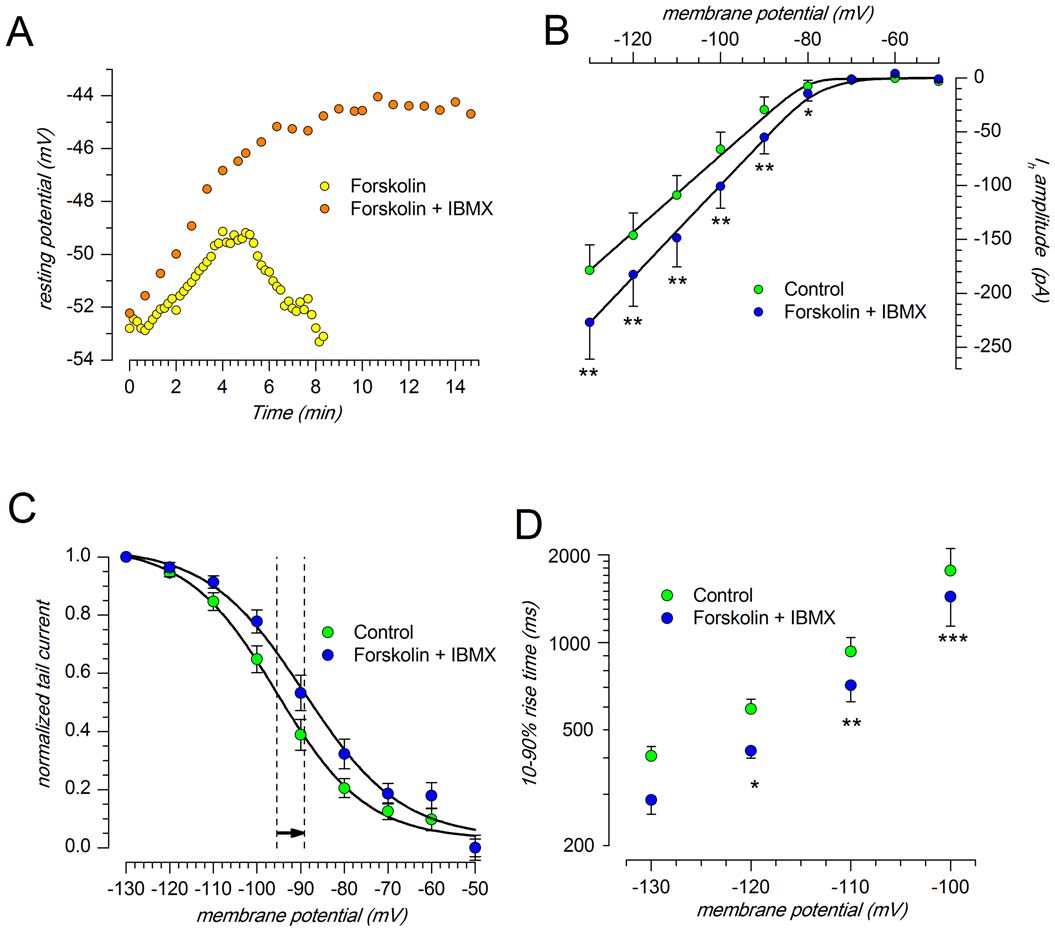
Effect of an increase of intracellular cAMP stimulated by forskolin on h-current. A: Effect of forskolin (10 µM) alone (yellow) and in association with IBMX (0.1 mM, orange) on resting membrane potential; explanation in the text. B: I/V relationship of the h-current in control conditions (green) and in the presence of forskolin+IBMX (blue); two-way ANOVA and Bonferroni post-hoc test: * indicates P<0.01, **P<0.001. C: Effect of forskolin+IBMX on steady-state activation curve. The shift of the midpoint in the depolarizing direction (6.33±0.68 mV, n = 8) is significant at the 0.0005 level. D: effect of forskolin+IBMX on the 10–90% activation rise time of the h-current; two-way repeated measures ANOVA and Bonferroni post-hoc test: * indicates P<0.05, **P<0.01 and ***P<0.001. All the experiments shown were performed at 37°C.

The first experiments were conducted in current clamp conditions to determine the effects of increased intracellular cAMP on the resting membrane potential. The addition to the extracellular solution of 10 µM forskolin, a classical activator of adenylyl cyclase [48], caused a depolarization of 3.5±1.3 mV (n = 5) after 4 min of application. Then the effect declined, vanishing completely in the next 5 min with a return to the resting membrane potential, even maintaining the forskolin supply (Figure 7A, yellow dots). However, when the bathing solution was further enriched with 0.1 mM IBMX, a phosphodiesterase inhibitor [49], the depolarization induced by forskolin was more prominent (7.9±1.8 mV, n = 5), and persisted as long as the application of the drug was maintained (Figure 7A, orange dots).

Under voltage-clamp conditions, the bath application of 10 µM forskolin and 0.1 mM IBMX, induce a significant increase of I_h_ amplitude (Figure 7B): at −130 mV the current amplitude is −178.5±23.5 pA in control conditions (n = 8), and −227.0±34.2 pA (n = 8) in the presence of increased levels of cAMP; at any tested potentials the increase in current amplitude was statistically significant (P<0.005 level, t-test for paired data).

The effect of forskolin on the h-current is twofold. First, it promotes a depolarizing shifts of the steady-state activation curve V_50_ in the depolarizing direction of 6.33±0.78 mV (n = 8; a variation significant at 0.0005 level; Figure 7C). In addition, following the increase of intracellular levels of cAMP, the I_h_ activation time course becomes significantly faster: from 1763±340 ms in control to 1435±298 ms for the 10–90% rise time (n = 8; p<0.025) at −100 mV (Figure 7D).

#### I_h_ modulation by endogenously released neurotransmitters

We next sought for possible modulation of the h-current in SNc neurons by endogenously released neurotransmitters acting on metabotropic receptors. Although modulation of I_h_ by endogenously released neurotransmitters other than dopamine has not been demonstrated yet, these effects could contribute significantly to the regulation of neuronal excitability, given the role of the h-current in controlling the resting membrane potential and ultimately the excitability profile of these cells. The effect of the principal neurotransmitters on the I_h_ was analyzed in perforated patch recordings at 37°C using high potassium concentration in the external saline in order to increase the amplitude of the current.

Of the dopaminergic receptors cloned so far, the D_2_ is the most abundantly expressed in the substantia nigra pars compacta neurons [50]–[52], where it is densely packed in the pericarya [53]. Since the activation of D_2_-like family receptors is coupled to a G_αi_ protein, which reduces the intracellular levels of cAMP by inhibiting the activity of the adenylate cyclase [54], we investigated whether the activation of D_2_ receptors had a modulatory effect on the h-current.

**Figure 8:**
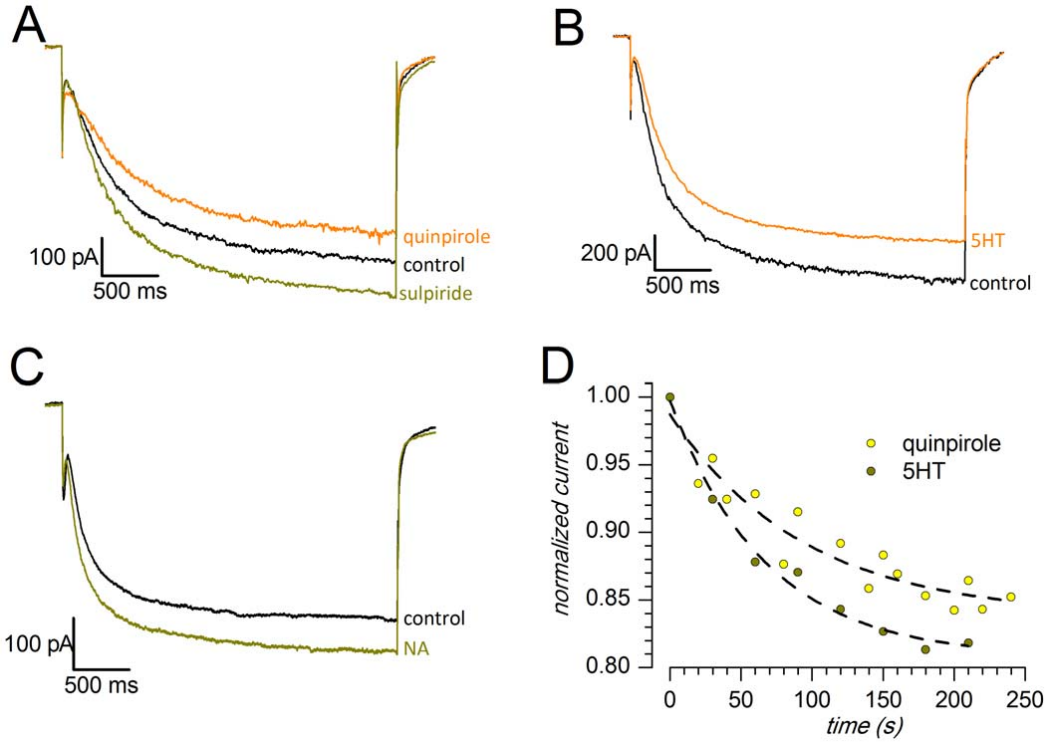
Sample tracings showing the effect of different neurotransmitters on the h-current; responses to hyperpolarizations to −130 mV from a holding potential of −40 mV; perforated patches, 37°C. The external saline included TTX, 4AP, TEA and Ba2+. A: Effects of the D2 agonist quinpirole (30 µM) and of the D2 antagonist sulpiride (20 µM); note that in the presence of the antagonist the response is greater than controls. B: Effect of 5-HT (100 µM). C: Effect of NA (100 µM). D: Time constant of the development of the effects of quinpirole and 5-HT.

In the presence of the D_2_-like agonist quinpirole (30 µM), the current amplitude was reduced (Figure 8A): at −130 mV, the measured current was −653.3±221.5 pA in control conditions (n = 6) and −557.0±189.2 pA in the presence of the agonist (n = 6), a difference significant at the 0.01 level (one-tailed *t*-test for paired data). On average, the D_2_-like agonist applied focally induces a 15% reduction of the h-current amplitude in about three minutes, with a time constant of about 93 s (Figure 8D).

The application of the D_2_-like antagonist sulpiride (20 µM) suppressed the effect of quinpirole, and caused a 16.5±2.34% increase in I_h_ amplitude above the control point (Figure 8A), increase significant at the 0.005 level (two tailed t-test for paired data n = 4); in other words, sulpiride not only quenched the effect of the antagonist, but resulted in an increase of the h current in the absence of exogenous D_2_ agonists. Since sulpiride is not an activator of adenylate cyclase, the effect can be explained by the presence, reported in this brain area, of a spontaneous release of dopamine at rest [55], whose action on autoreceptors would result in a tonic inhibition of the adenylate cyclase.

Serotonin (5-HT) is a critical neurotransmitter in the generation and regulation of emotional behavior and plays a prominent role in the inhibition of impulses. Substantia nigra receives serotoninergic input via fibers from the dorsal raphe nucleus [56]–[58], and 5-HT containing terminals make direct contacts over DA-containing neurons [59].

In midbrain DA neurons, serotonin has been reported to inhibit I_h_ in the VTA dopaminergic neurons in concentration-dependent manner [60], and to enhance the current in the substantia nigra pars compacta [61]. Therefore, we examined whether the 5-HT (100 µM) had any effect on the h-current performing the experiments in perforated patch-clamp configuration at 37°C. Using a single-pulse protocol from −40 mV to −130 mV, following application of serotonin and in the presence of Ba^2+^ to avoid collateral effects on the K_IR_ current (see discussion), we observed a reduction of the h-current amplitude in 5 out of 8 cells (Figure 8B), whereas in the remaining cells we detected no significant changes (3 cells). In the first group the reduction in I_h_ amplitude was about 20%, and was significant at the 0.05 level (*t*-test, single queued). The progression of the effect in the time domain is shown in Figure 8D - the time constant measured was of 67 s.

A diffuse network of noradrenaline (NA)-containing nerve endings in the neuropil of SNc has been demonstrated. The noradrenergic input is from the locus cœruleus [62]–[64] and -to a lesser extent- from other NA-containing neurons in the brainstem [65], [66]. NA modulates the h-current in several type of neurons, among which thalamic relay neurons [67], neurons of the medial nucleus of the trapezoid body (MNTB) [68], dorsal root ganglia neurons [69] and VTA neurons [70], [71]. In the thalamus, NA, acting on β-adrenergic receptors, increases the intracellular levels of cAMP and shifts the I_h_ steady-state activation curve to more depolarized potentials, as it does in MNTB neurons. On the contrary, activation of α_2_ adrenergic receptors in the DRG and VTA neurons causes a significant reduction in amplitude of I_h_.

We therefore tested whether the NA (100 µM) could determine any change in the h-current of DA neurons of SNc. We performed experiments in perforated patch configuration at 37°C, applying a single hyperpolarizing pulse from −40 mV to −130 mV. Upon application of NA, in 8 out of 10 cells we observed an increase in the amplitude of the h-current by about 11.8±1% (Figure 8C), whereas no change was detected in the remainder of SNc cells. The increase in amplitude was statistically significant, with a P-value <0.025 using single-tailed t-test for paired data.

## Discussion

Two classes of hyperpolarization-activated inwardly rectifying currents have been reported in SNc neurons. One type has fast kinetics, is permeable primarily to K^+^, is blocked by extracellular Ba^2+^ and Cs^+^, and has a voltage-dependence that is itself dependent on extracellular K^+^ ([K^+^]_o_) concentration. I_h_ (or I_f_ in cardiac tissue), the second type of inward rectifier, is a mixed cation current, with a reversal potential substantially positive to E_K_ [72]. I_h_ has a relatively slow activation kinetics, is insensitive to Ba^2+^, and does not show a voltage sensitivity dependent on [K^+^]_o_ [25]. The sensitivity to drugs very selective for I_h_ like ivabradine or ZD7288, the Ba^2+^ insensitivity, the slow kinetics of activation and the reversal potential suggest that the current described here belongs to the latter class.

From the methodological point of view, a first novelty of this study is in the use of a transgenic line of animals that expresses a reporter protein (eGFP) under the TH promoter, allowing the precise recognition of DA neurons in *in* *vitro* slices. This is of some importance as, in these preparations, neurons are often identified as DAergic according to the expression of I_h_, a current which is not -or much less- expressed in putative non-DA neurons [1], [9], [73], [74]. Notably, while the absence of I_h_ in a DA neuron is a reliable predictor that the cell is not DA-containing, the presence of I_h_ does not reliably predict TH co-labeling [16], [17]. In other words, since a significant number of neurons expressing the h-current is not DAergic, this means that one of the principal markers assumed to identify midbrain DAergic neurons is not associated exclusively, or even significantly, with confirmed DAergic neurons. The identification of the neurons by the expression of eGFP under the TH promoter, marks a first important difference of this study with respect to other functional studies, where the expression of TH (when done) was carried out only in few sample cells.

A second methodological hallmark of this work is the choice of working systematically at 37°C and in perforated patch configuration, especially when the modulation of the h-current was studied. These working conditions are more respectful of the intracellular milieu and, in our view, lead to significant differences in the observed results.

In this preparation, Ivabradine was used for the first time as a blocker of I_h_. This drug, originally developed ad a bradicardic agent [44], proved to be an excellent blocker of all types of HCN subunits [37], [75]. The reason of the preference accorded to this drug resides in the rapidity and complete reversibility of its action, whereas the more classical I_h_ blocker ZD7288 is slower and often irreversible [76], [77].

### Biophysical Properties

#### Steady-state activation

At 37°C, and a 4 s hyperpolarizing pulses, we found a midpoint of activation at −82.73±1.17 mV with a slope of 7.29±0.28 mV (n = 12); however, as first shown by ref. [18], the point of half-activation depends critically on the used voltage protocol. In particular, short voltage steps do not allow channel activation to come to completion and then the steady-state activation curve derived from tail current amplitudes is seriously underestimated. Using different pulse duration, and extrapolating to the asymptote the trend of the midpoint shift in the depolarizing direction as a function of the hyperpolarizing pulse duration (Figure 1D), we calculated a V_50_ of −77.05 mV, i.e. from 10 to 28 mV more depolarized than the values reported in SNc neurons at 37°C [9], [78].

The functional implication of these values becomes obvious if one considers that assuming a slope of 7.43 (the slope is 7.41, 7.36, 7.29 and 7.65 at 1, 2, 4 and 8 s, respectively), at −65 mV about 16.5% of the h-channels are open, which corresponds to a conductance of 1.023 nS. Since we found an average input resistance of these cells of 414 MΩ, at −65 mV the h-current gives a contribution of +8.9 mV to the resting membrane potential, a value matching very well the hyperpolarizations found upon blocking the h-current with ivabradine in the present experiments (−11.83±2.07 mV). Such an hyperpolarization would be impossible with the kinetic coordinates reported in most of the previous papers and the value reported in literature. Since the hyperpolarizing effect of the I_h_ blockage is controversial, we repeated the experiments using the more classical blocker ZD7288 (30 µM), obtaining results substantially similar to those obtained with ivabradine, although the hyperpolarizing effect was less pronounced (−6.87±0.78 mV, n = 15).

There could be several explanations to justify the discrepancy of our data with others reported in literature, which do not show any effect of the h-current on the resting membrane potential [9], [12]–[15], [79].

First, it cannot be excluded that ivabradine could also act on conductances different from the h-current. In fact, a inhibitory effect of high concentrations of ivabradine on L- and T-type calcium currents [44], [45] has been reported; however, at the concentrations used in the present experiments (10 µM), to the best of our knowledge, no side effects have ever been described, although these cannot be certainly excluded, considering the still scarce knowledge of this drug in nerve cells. However, we see a hyperpolarizing effect also of ZD7288; furthermore, notice that our result (a hyperpolarization of 6.9 mV) is almost coincident with that reported in a recent paper (7 mV; [80]).

Second, as shown by previous studies, I_h_ amplitude correlates with calbindin (CB) expression in nigral dopamine cells, with larger currents in CB-negative cells [13], [81], and dissimilar proportions of CB-positive and CB-negative cells could have been analyzed in different studies - in this regard, direct analysis by molecular methods would help to characterize calbindin expression when recording from SN neurons.

#### Temperature dependence

For what concerns current amplitude, the Q_10_ values reported by various authors lie in the range between 1.35 [20], [82] and 6 [83], with an average around 3. We found a value of 3.76±0.53, which is well aligned with most of the reports. This increase is generally ascribed to depolarizing shifts in the steady-state activation curve for the h-current which, for a 10°C temperature increase, range from 2–3 mV [82] to 12–13 mV [31], [84] without change in the slope. In SNc neurons, rising the temperature from 27 to 37°C we observed a depolarizing shift in the midpoint of the steady-state activation curve (V_50_) of 10 mV in the depolarizing direction, without any change in the slope.

The main difference in temperature sensitivity with respect to the current literature was observed in the activation rise time, for which we found a Q_10_ of 6.4, while values found by other authors are around 3 [30], [34], [82] - a diversity that can be explained by differences in the preparation and consequently in the subunit composition of the channels.

The high temperature-dependence of the I_h_ in SNc neurons underscores the importance of controlling this often neglected parameter in the electrophysiological recordings of this current. This dependence can be at the basis of many of the discrepancies found in literature.

The high sensitivity of the h-current in substantia nigra neurons might at least in part explain the previously observed warming-induced increase in firing frequency, decrease in input resistance, and an inward current reversing its polarity between −5 and −17 mV, which is dependent on extracellular Na^+^ [85]. In this context, it might also be useful to recall that changes of up to several degrees centigrade in the brain temperature are observed not only during fever, but also during different behavioral states [86]. Furthermore, neurons in different brain regions, including the substantia nigra, were reported to show high temperature sensitivity [87], a behavior whose underlying mechanism is unknown. Our data indicate that the h-current might have a role in the process.

### Pharmacological Properties

The fundamental involvement of I_h_ in the control of resting membrane potential makes the h-channel a privileged target for neurotransmitter systems aiming at the regulation of SNc neurons activity. In general, the excitability profile of a cell expressing an h-current can be controlled either by moving the membrane potential in and out of the range of I_h_ activation, or by moving the I_h_ activation within the range of membrane potentials of physiological interest by directly modulating the h-channel itself. In fact, a number of intracellular signaling molecules can affect I_h_ in SNc neurons, including -besides the cAMP- also phosphoinositides [88], [89] and kinases [90]. In particular, the possibility to control the voltage dependence of the h-channels in SNc neurons appears to enable transmitters of ascending activation systems, such as serotonin, noradrenaline, and dopamine, to specifically reduce or increase the membrane impedance, and more in general the excitability profile of the cell. Some of these influences, like modulation by GABAergic input arising from other nuclei in the basal ganglia, have been carefully studied (for a review see [91]), other, as those mediated by cyclic nucleotide pathways, have been less extensively investigated. In this work we have attempted a preliminary exploration of the possible modulation of the h-current by some neurotransmitter known to act on SNc neurons.

One known possible source of error is that the modulation (inhibition) of the h-current occurs secondary to GIRK channel activation [9], [78], [92], since between GIRK and I_h_ channels there is a close link [93], [94]. We can exclude this possibility since, in experiments where the modulation of the h-current was studied, we were always in the presence of Ba^2+^ at concentrations that can entirely block the inward rectifier K^+^-currents, GIRK included.

#### I_h_ modulation by cAMP

The h-channels are directly activated by cyclic nucleotides [46], but when tested in SNc neurons, the adenylate cyclase activator forskolin gave contradictory results on the amplitude of the h-current, going from a complete absence of effect [78] to a 25% increase, due to a +5.33 mV shift of the steady-state activation curve towards more positive potentials [95]. Our results are almost exactly superimposable with those of the latter authors, with a 27.5% increase in current amplitude and a +6.33 mV shift of V_50_. It should be noted that initially we were unable to see any influence because we tested the forskolin effect 10 minutes after application, and without IBMX. In these conditions, the modification of the resting membrane potential is transient and vanishes after 7–8 min (Figure 7A), but in the presence of the phosphodiesterase inhibitor the effect is larger and stable in time, and this might explain some discrepancy in the literature about this point.

#### D2 receptors

Since its discovery by Aghajanian in 1977 [96], [97], the presence of D2 receptors in midbrain DA neuron, is considered one of the hallmarks of these cells, and as such it has received much attention. DA D2-like receptors are present as auto-receptors on the DA neurons in SN and VTA and play an important role in the regulation of DA neuronal firing activity by means of auto-inhibition (for a review see [98]. These G protein-coupled receptors are activated by dendritically released DA [99], [100] through a still controversial mechanism [101], and the hyperpolarization following their activation is classically described as mediated through an activation of potassium channels, GIRK type [43], [78], [102], and A-type [103], [104]. In the present work, we show that, in addition, also the h-current is modulated by the activation of D2-like type DA receptors. This is not surprising, as D2 receptors are coupled to a G-protein G_αi_ which directly inhibits the formation of cAMP by inhibiting the enzyme adenylate cyclase [54]. Our observation confirms a relatively recent study, conducted in brain slices at 34°C showing that DA, released endogenously following a single action potential, hyperpolarizes neighboring DA neurons by inhibiting h*-*channels [105].

Thus, it is possible that DA autoreceptors are linked to different effector systems. Receptors that are coupled to h-channels may be preferentially located in the vicinity of DA release sites, whereas those coupled to G-protein-gated K^+^ channels may be extrasynaptic.

#### Serotoninergic receptors

The bulk of available neuroanatomical data clearly indicate that the midbrain DA- neurons receive a prominent innervation from 5-HT originating in the raphe nuclei of the brainstem [106]–[108]. The modulatory action of 5-HT on midbrain dopaminergic neurons is complex, and probably exerted through different pathways (for an overview see [109], [110] - here we have limited the analysis of the 5-HT to its effects on the h-current.

In general, 5-HT is reported to increase the h-current in the CNS [67], [111], [112]. In the midbrain DA neurons both enhancement [61] and inhibition [60] of the I_h_ have been reported. Our data confirm the first action, although we cannot establish whether the effect is direct or indirect.

#### Noradrenergic receptors

Controversial data were reported concerning the effects of NA in SNc. Several studies indicate that electrical stimulation of the locus cœruleus evokes an initial excitatory response in SNc neurons, frequently followed by a period of inhibition of firing [64], [113]. *In vivo* studies report an hyperpolarization in rat SNc neurons by NA [43], which slows the frequency of spontaneous action potentials [114]. In SNc neurons other authors have found that NA apparently induced an inhibition of I_h_, but then they report that in the presence of 300 µM external barium or internal cesium, NA did not affect I_h_, suggesting that the effect on I_h_ is secondary to the activation of K_IR_ channels [92]. Contrary to what was found by these authors, we can exclude an indirect effect mediated by K_IR_ channels as we were systematically recording in barium 500 µM. We did not look for better specification of the pathway involved in the NA stimulation of the h-current, which Cathala and Pupardin-Tritsch suggest being PKC, nor of the receptor involved, for which both α1 and α2 adrenergic types seem to be excluded [92].

Nevertheless, the observation of Cathala and Pupardin-Tritsch that NA inhibits the K_IR_ current, in addition to enhance the I_h_, is rather interesting, as there is an increasing evidence that also in other systems amine-activated pathways can modulate both I_h_ and K_IR_ acting in opposite direction. A synchronous and symmetrical control of the balance between I_h_ and I_KIR_ was also described for example in rat spinal motoneurons, where 5-HT increases the cell excitability inhibiting an I_KIR_ and enhancing an I_h_ [115]; in salamander motoneurons, where muscarinic modulation inhibits the I_h_ and enhances the I_KIR_ [116]; in DAergic neurons of the olfactory bulb, where cAMP increases the I_h_ and inhibits the I_KIR_ (personal unpublished observation).

In SNc neurons, where I_h_ is enhanced and I_KIR_ is inhibited by NA, this synchronous and symmetrical modulation might underlay a mechanism that could shorten hyperpolarizing events. Since the activation and deactivation kinetics of I_KIR_ are much faster than that of I_h_, the net result would be a sharper response of the neuron in response to hyperpolarizing events. The enhancement of I_h_ by NA could then accelerate the recovery from hyperpolarizations, ultimately shortening their duration and increasing firing frequency, a combined action that might be shared, not necessarily with the same sign, also in other systems.

### Functional Implications and Conclusions

A substantial amount of research has focused on determining the factors that alter the activity of substantia nigra DAergic neurons. Much of this research indicated that several mechanisms that regulate dopamine neuron activity have the capability to maintain the baseline activity of dopamine cells at a fairly constant rate [117]. However, DAergic cell activity can be finely tuned also by afferent inputs that involve an assortment of ionotropic and metabotropic receptors acting directly on the neurons themselves. In addition, the neurons can modulate the input received at local level through dendritic dopamine release that affect their own responsiveness to afferent input by controlling dendritic excitability through D2 autoreceptors. We show that the I_h_, with its role in the control of membrane potential, seems to be an important target of the afferent inputs to SNc neurons. We propose that this current may be one of the main actors responsible for the rich signaling repertoire displayed by these cells which, through their effects on forebrain dopamine levels, influences much of the functioning of the basal ganglia as a whole.

## Materials and Methods

### Animals and Surgical Procedures

Experimental procedures were carried out to minimize animal suffering and the number of mice used. The procedures employed were in accordance with the Directive 86/609/EEC on the protection of animals used for experimental and other scientific purposes, and were approved by the Campus Veterinarian of the Ferrara University. A total of 192 mice have been used, most of them in the 14–20 postnatal day range, and 20 over 3 months old. All experiments were performed using the transgenic mice TH-GFP/21–31 line carrying the eGFP gene under the control of the TH promoter [118], [119]. Transgenic mice were identified either by PCR on the genomic DNA extracted from tail biopsies, or -at postnatal day 3 or 4- looking at the fluorescence of the olfactory bulbs transilluminated with a UV source (FBL/Basic-B & N-01; BLS, Hungary; FHS/F-01) and observed with an emission filter (FHS/EF-2G2; BLS, Budapest, Hungary). Transgenic lines were maintained as heterozygous by breeding with C57BL/6J inbred mice.

### Preparation of Midbrain Slices

Mice were anaesthetized (intraperitoneal injection of 60 mg/kg of sodium pentobarbital) and decapitated. The brain was removed from the skull in less than 1.5 min and put into ice-cold (2–4°C) dissection solution of the following composition (in mM): 3.0 KCl, 1.25 NaH_2_PO_4_, 2 MgCl_2_, 1.6 CaCl_2_, 10.0 glucose, 21.0 NaHCO_3_, 215 sucrose; saturated with 95% O_2_ and 5% CO_2_.

A brain section containing the substantia nigra pars compacta was obtained as follows. Two coronal cuts were performed, the caudal to remove the cerebellum and the rostral half of the cerebral hemispheres. The resulting block was glued on rostral surface with n-butyl cyanoacrylate adhesive (3 M™ Vetbond™, Segrate, Italy) to the support of vibratome (Campden HA 752, Loughborough, England) and then submerged by ice-cold dissection solution. The coronal slices (thickness, 150 µm) were cut starting from the caudal surface. Slices containing the substantia nigra pars compacta were identified by illuminating the specimen with a desk UV light source (BLS), stored in an incubation chamber containing artificial cerebrospinal fluid (ACSF) continuously bubbled with carboxygen (95% O_2_, 5% CO_2_), and then kept at room temperature for about 10 hours.

Slices were placed in the recording chamber and superfused with ACSF at a rate of 2 ml/min.

### Current and Voltage Recordings

The temperature of the 1-ml recording chamber was controlled using a couple of 39.7 W Peltier devices (RS Components, Milan, Italy) and measured with a high-precision, low mass thermocouple (RS Components, Milan, Italy).

Current and voltage recordings were acquired with an Axopatch 200B amplifier (Molecular Devices, Sunnyvale, CA), and a 12 bit A/D–D/A converter (Digidata 1440A; Molecular Devices); the holding potentials were corrected for the junction potential, calculated using the related function of the acquisition software (pClamp 10, Molecular Devices). Borosilicate glass pipettes (1.5 o.d., 0.87 i.d., with filament; Hilgenberg, Malsfeld, Germany) were pulled with a Zeitz-DMZ puller (Martinsried, Germany) and had a resistance of 4–5 MΩ when filled with standard intracellular (IC) solution; the seal formation was realized with the help of an air pressure controller (MPI, Lorenz Messgerätebau, Katlenburg-Lindau, Germany); the seal resistance was always greater than 3 GΩ. A 70–80% compensation of the series resistance and correction for junction potential was routinely used.

### Solutions

The solutions used had the following composition (mM):

*standard ACSF extracellular (EC) saline*: 125 NaCl, 2.5 KCl, 26 NaHCO_3_, 1.25 NaH_2_PO_4_, 2 CaCl_2_, 1 MgCl_2_, and 15 glucose;

*high K EC solution*: 115 NaCl, 12 KCl, 26 NaHCO3, 1.25 NaH_2_PO_4_, 2 CaCl_2_, 1 MgCl_2_, and 15 glucose.

*high sucrose EC dissection solution*: 3.0 KCl, 1.25 NaH_2_PO_4_, 2 MgCl_2_, 1.6 CaCl_2_, 10.0 glucose, 21.0 NaHCO_3_, 215 sucrose.

All EC solutions were continuously bubbled with 95% O_2_ and 5% CO_2_; the osmolarity was adjusted at 305 mOsm with glucose.

*standard pipette-filling intracellular (IC) solution*: 120 KCl, 10 NaCl, 2 MgCl_2_, 0.5 CaCl_2_, 5 EGTA, 10 HEPES, 2 Na-ATP, 10 glucose. The free calcium concentration with this internal solution was calculated to be 16 nM (http://www.stanford.edu/~cpatton/downloads.htm).

For perforated patches, amphotericin B was included in the recording electrode filling solution as perforating agent (200 µg/ml plus 300 µg pluronic F-127). In order to make sure of the integrity of the perforated patch, EGTA was omitted from this solution and the concentration of CaCl_2_ was raised to 3 mM. Data were collected after the series resistance fell to <50 MΩ.

In all IC solutions the osmolarity was adjusted to 295 mOsm with glucose, and the pH to 7.2 with KOH.

Ivabradine was a generous gift from Servier (Suresnes, France).

### Analysis of Current Recordings

Offline analysis was performed using version 10.2 of pClamp (Molecular Devices) and version 8 of Origin (OriginLab Corporation, Northampton, MA).

The I_h_ amplitude was measured as the difference between the steady-state current at the end of test voltage pulses (I_ss_) and the instantaneous current and the beginning (I_inst_); the latters were measured extrapolating the exponential fitting the h-current (single or double, see below) to the time of the onset of the hyperpolarizing pulse, as indicated by the arrow in Figure 4E.

Rates of I_h_ activation were determined using the following function (Clampfit 10.2, Molecular Devices):
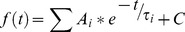
(3)where *i = *1 or 2 (a single or double exponential fit), *A* is the amplitude of the fitting component(s), *τ* is the time constant, and *C* is the shift of the fitted trace from zero.

The activation curve of I_h_ was constructed using a two-step protocol [120]: the I_h_ was first activated to a variable degree by a conditioning step, and then fully activated by a second pulse to −130 mV (Figure 1B). The resulting tail current amplitudes were then normalized and fitted by the equation:



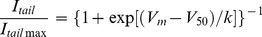
(4)where I_tail_ is the amplitude of the tail recorded at the second pulse, I_tailmax_ is the maximal amplitude of the tails, V_m_ is the membrane potential; V_50_ is the membrane potential at whom half of the channels are open (midpoint); *k* (slope) represents the dependence of channels opening from the change of potential.

The temperature coefficients of activation and deactivation time constant were defined as:
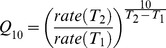
(5)thus, for every 10°C of change in temperature there is a *Q*_10_-fold change of the rate analyzed.

Unless otherwise stated, data are presented as means ± s.e.m. Statistical significance of the results was assessed with one-way or two-way analysis of variance (ANOVA), or Student’s *t* test for paired samples, as indicated; the software used was Prism 5 (GraphPad software, Lajolla, CA). A *P* value *<*0.05 was considered significant.

## Supporting Information

Code S1 **Matlab code for the solution of** **eqn. 3** **.** (Word file).
(DOC)
