# Supplementary material for: The h-Current in the Substantia Nigra pars Compacta Neurons: A Re-examination
Source: PLoS One. 2012 Dec 21;7(12):e52329. doi: 10.1371/journal.pone.0052329 (PMC3528748; doi:10.1371/journal.pone.0052329)
Supplement: Code S1 — Matlab code for the solution of eqn. 3 . (Word file). (DOC) [file pone.0052329.s001.doc]

Matlab code for the solution of eqn. 3:

% Parameters

A1 = 23.41;

A2 = 100 - A1;

tau1 = 3402;

tau2 = 746.64;

y = 10; % value of the function: set y=10 and y=90 to compute the time at

% which the function has had a decrement of 90 and 10 percent,

% in this order, respectively

a = 10; b = 6000; % lower (a) and upper (b) limit of the interval within

% which the solution is expected to be found

% Definition of the function f(t) of the equation in the form f(t) = 0

f = @(t)( A1*exp(-t/tau1) + A2*exp(-t/tau2) - y );

% Search of solution within the interval [a,b]

disp(sprintf('\nf(t) = A1*exp( -t / tau1 ) + A2*exp( -t / tau2 ) - y'));

disp(sprintf('A1 = %f A2 = %f y = %f',A1,A2,y));

disp(sprintf('\nSoluzione dell''equazione f(t) = 0 in [%f,%f]',a,b));

ts = fzero(@(t)f(t), [a b]);

% Visualiztion of the graph of f(t) and record of the figure

% in two files in format .pdf and .png

tt = [a:0.1:b]';

yy = f(tt);

plot(tt,yy,'b-',[tt(1) tt(end)],[0 0],'k-');

xlh = xlabel('t');

ylh = ylabel('f( t ) = A_1 exp( - t / \tau_1 ) + A_2 exp( - t / \tau_2 ) - y');

tlh = title(sprintf('A_1 = %6.3f, A_2 = %6.3f, y = %6.3f',A1,A2,y));

hold on;

plot(ts,0,'+r','LineWidth',1);

axis('tight');

ylim = get(gca,'YLim');

th = text(ts,+0.05*(ylim(2)-ylim(1)),sprintf('t^* = %6.3f',ts),'HorizontalAlignment','Left');

hold off

set([xlh;ylh;tlh;th;gca],'FontName','Times');

print -dpdf -r300 figura.pdf

print -dpng -r300 figura.png
